# Deterministic Creation of Macroscopic Cat States

**DOI:** 10.1038/srep13884

**Published:** 2015-09-08

**Authors:** Daniel Lombardo, Jason Twamley

**Affiliations:** 1 Centre for Engineered Quantum Systems, Department of Physics and Astronomy, Macquarie University, Sydney, NSW 2109, Australia

## Abstract

Despite current technological advances, observing quantum mechanical effects outside of the nanoscopic realm is extremely challenging. For this reason, the observation of such effects on larger scale systems is currently one of the most attractive goals in quantum science. Many experimental protocols have been proposed for both the creation and observation of quantum states on macroscopic scales, in particular, in the field of optomechanics. The majority of these proposals, however, rely on performing measurements, making them probabilistic. In this work we develop a completely deterministic method of macroscopic quantum state creation. We study the prototypical optomechanical Membrane In The Middle model and show that by controlling the membrane’s opacity, and through careful choice of the optical cavity initial state, we can deterministically create and grow the spatial extent of the membrane’s position into a large cat state. It is found that by using a Bose-Einstein condensate as a membrane high fidelity cat states with spatial separations of up to ∼300 nm can be achieved.

Observation of quantum mechanical effects on large scale systems has been intensively pursued since the development of quantum mechanics. This is because such observations would demonstrate the existence of quantum mechanical states which reside at the borders of the classical and quantum worlds. The creation of such ‘macroscopic’ quantum states has been a long sought after goal in quantum mechanics allowing not only for the direct study of quantum state collapse models^1^ but for their extension to potentially practical applications in quantum information, quantum metrology and quantum simulation^2^,^3^,^4^,^5^,^6^,^7^.

Many approaches have been made to observe quantum mechanical effects on larger scales. In particular, extensive efforts have been direct towards up-scaling the well-known two-slit experiment in hope to observe wave like behaviour of objects much larger than electrons. To date, wave like behaviour has been reported with the use of particles ranging from single atoms all the way to organic molecules containing several hundreds of atoms^8^,^9^,^10^. The creation of quantum superpositions and entangled states of trapped macroscopic objects is also widely sought after. Of particular interest is the creation of states which are analogous to the famous Schrödinger’s cat state due to both their simplicity and their completely quantum mechanical nature. In quantum optics photonic forms of the Schrödinger’s cat state are also studied. These states are commonly referred to as ‘cat states’ and often correspond to superpositions between two near-orthogonal coherent states of the electromagnetic fields in cavities. Both Schrödinger and superposition cat states have been observed by either entangling the internal state of an atom with its position^11^, or with a coherent light field before performing a measurement^12^. However, the largest of these corresponds to an entangled spatial superposition of a single ion where the spatial displacement between the two ion positions was 83 nm^11^. More recent are proposals for matter-wave interferometery by a falling nanoparticle^13^, experimental demonstration of matter-wave interference for high-mass molecules^14^, and proposals for space-born macroscopic quantum resonators for testing quantum mechanics^15^. Many of these proposals/experiments produce a quantum state whose spatial superposition is entangled (correlated), with other ancillary degrees of freedom (e.g. internal spin states), and thus have a number of drawbacks. Firstly they suffer greater amounts of decoherence through coupling to other types of noise via these ancillary degrees of freedom and if one wishes to perform coherent experiments (e.g. interferometry), with entangled cats one must control both the spatial and ancillary degrees of freedom of the superposition to great precision. In what follows we aim to synthesise a *pure cat*, where the spatial superposition is completely disentangle from other degrees of freedom.

With the intention of creating macroscopic quantum states many researchers have directed their attention towards the field of optomechanics. Some of the more common optomechanical systems which are studied involve cavities that are comprised from two mirrors, one fixed and one moveable. The radiation pressure force due to the light confined within the cavity causes the second mirror to move, acting as a mechanical harmonic oscillator. Other types of optomechanical systems consider placing mechanical oscillators within Fabry-Pérot type optical cavities. Such systems have inherited the name “Membrane in the Middle” where the mechanical oscillator is referred to as the membrane^16^. Several types of membranes have been used in such setups, ranging from solid silicon nitride crystalline, flexible 2D films, through to Bose-Einstein condensates (BEC)^17^,^18^,^19^. There are many possible variations of these optomechanical setups which have been both studied and used as a platform for cat state creation^1^,^20^,^21^,^22^,^23^,^24^,^25^,^26^. Of these, perhaps the most macroscopic proposed involves a tiny mirror, consisting from 10^14^ atoms, which acts as a mechanical oscillator. This setup can achieve spatial separations on the order of a few femtometres with demanding experimental conditions^20^.

There is one, yet to be mentioned, limiting factor which hinders almost all of the current cat state creation schemes: they all involve measurement and thus are probabilistic. This is a severe drawback as the overall probability for success of the protocol exponentially decreases with the size of the cat state desired^27^. In this work a novel, completely deterministic method of creating cat states of the position of a macroscopic object will be proposed. These states will be created by exploiting properties in the optomechanical Membrane In The Middle (MITM) setup^16^. It will be shown that by controlling the membrane’s opacity its displacement can be driven at a rate proportional to the number of photons in the system. This will be achieved by effectively switching the membrane’s opacity between a reflective and a transparent state, as shown in Fig. 1. This technique will be used as a mechanism to create and deterministically grow the spatial extent of a quantum cat state. Before a cat state is produced, however, to ensure that the lifetime of the state is not limited by the finesse of the optical cavity the scheme requires disentanglement of the membrane’s final position from the two cavity modes. Disentangling such a state using the MITM model alone is extremely challenging experimentally, essentially requiring the membrane’s opacity to be also spatially dependent. Instead, an alternative deterministic disentanglement protocol will be proposed as the final step in creating the cat state. As a means of illustrating the cat state creation protocol a schematic experimental setup using optical light will be discussed . Finally, using a BEC type membrane, it will be shown that the proposed scheme is capable of creating high fidelity cat states with spatial displacements of up to ~300 nm using only a small photon occupation.

## Results

### The Membrane in the Middle Model

Our goal is to discover a deterministic protocol for the creation of a cat state of a mechanical object’s position. As mentioned above one approach which has been previously studied involves the introduction of mechanical oscillators into Fabry-Pérot type cavities (MITM). Here a similar approach will be used but now we also consider controlling the mechanical oscillator’s opacity. We first describe the MITM model and the light-matter interactions. Next, the effects of controlling the opacity of the membrane will be studied where two extreme cases will be considered. These cases correspond to either a reflective or a highly transmissive membrane. Finally, it will be shown that by alternating between these two opacity states the spatial displacement of the membrane can be driven.

### Introduction to MITM

**Figure 1:**
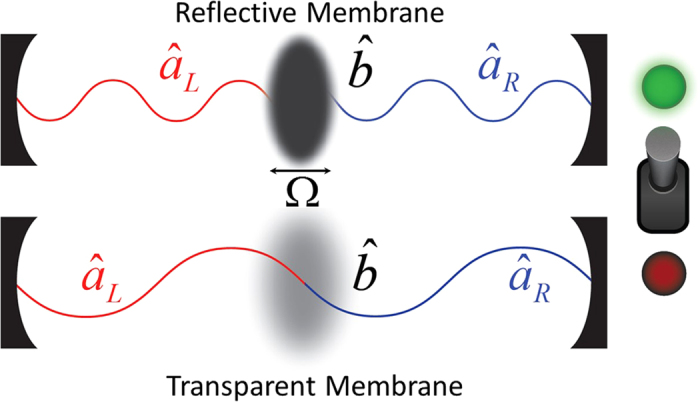
A depiction of the Membrane in the Middle setup is shown in two separate cases. The top cavity contains a reflective membrane while the bottom contains a transmissive membrane. The switch represents the control over the membrane’s opacity, where ‘on’ corresponds to a reflective membrane and ‘off’ to a transmissive membrane.

The Membrane in the Middle model describes an optomechanical system which is comprised from two degenerate modes of a cavity which interact with a physical membrane that is confined within the cavity, shown in Fig. 1. The Hamiltonian for this model can be split into four main portions. These include; 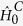, describing the self energies of the cavity modes, 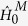, that of the membrane’s motion, 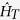, which describes the transmission of light through the membrane and 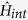 the interaction energy. The self energy terms are given by,


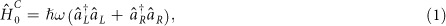



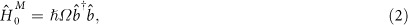


where 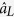/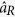 denote the annihilation operators of the left/right cavity modes with frequency 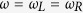 and 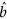 that of the membrane’s mechanical motion with frequency Ω. In what follows optical frequency modes will be focused on, for practical purposes. The transmission term describes the transfer of photons between the left/right cavity modes through the membrane. It can be described by,


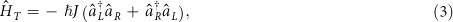


where *J* is the transmission rate of the membrane. Finally, we include the interaction term describing the photon pressure force of the cavity mode photons acting on the membrane. This interaction requires that the wavelength of the optical modes satisfies 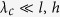, where *l*/*h* represent the membrane’s length/height. The photon pressure force can be expressed as 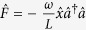, where 
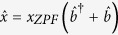
 is the position operator of the membrane, 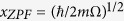 the zero point fluctuation amplitude and *L* is the length of the individual cavities. The optomechanical interaction term is then expressed as,


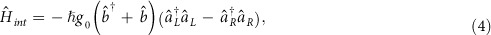


where 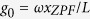 is the optomechanical coupling strength. The Hamiltonian of the entire system is now 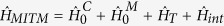. By noticing that 
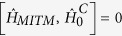
 this Hamiltonian can be reduced in the interaction picture of the cavity modes to give,


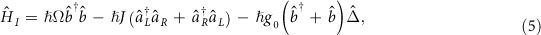


where the difference operator 
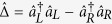
 has been introduced.

### Controlled Transmission in the MITM Model

With the theory behind the MITM model established the effects of controlling the transmission rate, *J*(*t*), can be studied. Two extreme cases of *J* can be considered. The first corresponds to a reflective membrane, in particular when 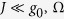. In this case 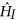 approximately reduces to,


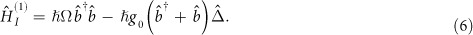


**Figure 2:**
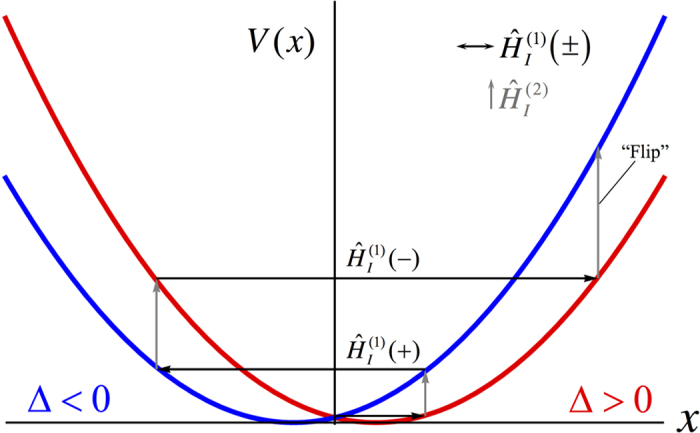
Classical visualisation of driving the membranes displacement by alternating the system between the two symmetrically displaced potential wells. The horizontal arrows represent the evolution of the membrane’s position in the high opacity regime where the membrane is reflective, 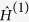, while the vertical arrows represent evolution in the transparent membrane regime, 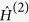, or, the flipping of the cavity states. The sign 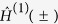 denotes the phase on the optomechanical driving term. Here the system is initialised in the state 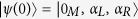 with Δ > 0.

This Hamiltonian describes a driven harmonic oscillator where the driving strength is directly proportional to not only the optomechanical coupling rate but also to the difference in the photon numbers between the optical modes. In the case where 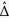 is treated as a classical number Eq. (6) corresponds to harmonic motion about a displaced position, which will be used to interpret later results. Under the evolution of 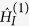 the difference in photon numbers between the left and right cavities is conserved, as 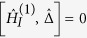, significantly simplifying the solutions to the Heisenberg equations of motion. These solutions show, ignoring dissipative effects, that when starting in an initial state 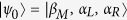, corresponding to the membrane initially in a coherent state *β*_*M*_ and the left/right cavity modes in coherent states *α*_*L*_/*α*_*R*_, the expectation value of the membrane’s position evolves as,


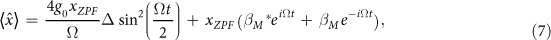


where 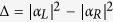. This expectation value shows that if the state of the left and right cavity modes could be completely interchanged at times satisfying 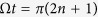, the position of the membrane could be driven to even larger spatial extensions, see Fig. 2. The reason for this is that interchanging the state of the left and right modes effectively switches the phase on the interaction term in Eq. (6), that is, 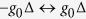. This results in the membrane experiencing an extra displacement of 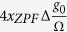 after every photon number interchange. One way to interpret the effect of the phase on the optomechanical driving term is to consider the direction of the membranes displacement. For example, if the system is initialised in the state 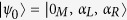 with Δ > 0 the phase on the optomechanical driving term is − and the membrane is displaced by 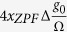 in the positive *x* direction. Alternatively, if the system is initialised in the state 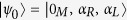 the phase on the driving term will be + and thus the membrane will be displaced by the same amount, but in the opposite direction. An alternative explanation can be made in the displaced harmonic oscillator picture to easily visualise the protocol described to increase the membrane’s maximal displacement. The separate ± phases of the optomechanical driving term correspond to two separate quadratic potentials which are symmetrically displaced about the origin, as shown in Fig. 2. Carefully timing the optomechanical phase switching to occur when the membrane has reached a maximal displacement in one harmonic potential is analogous to shifting the membrane to the other, displaced, harmonic potential, where the potential energy is larger. If this process is repeated by switching the membrane between the two symmetrically displaced harmonic potentials its energy can sequentially be increased to achieve larger and larger spatial displacements, see Fig. 2. The interesting feature is that since the two harmonic potentials are identical the switching times are independent of the spatial extent of the membrane.

In order to drive the displacement of the membrane a method of interchanging the state of the left/right cavities must next be established. As this typically requires the flow of photons through the membrane, the second limiting case of *J* will now be considered. That is, the case of an almost completely transparent membrane. In this case, as 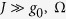, the transmissive term in Eq. (5) dominates and the Hamiltonian approximately reduces to,


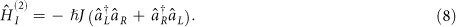


By again working in the Heisenberg picture the cavity mode operators can be evolved to give,


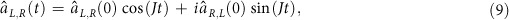


which shows that at times satisfying 
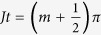
 the left and right modes can be interchanged completely. Using the initial state 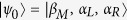 the expectation value of the mode number operators can now be evolved to give,


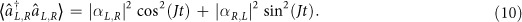


**Figure 3:**
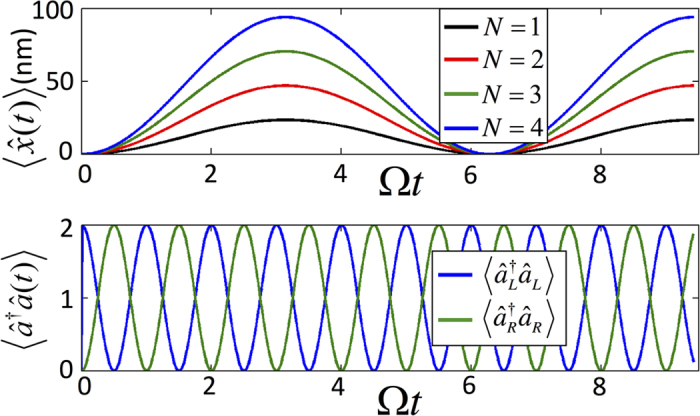
The dynamics of the MITM model in both the reflective membrane regime (a) and transparent membrane regime (b). Both results were produced via evolution of the initial state 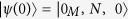 for a BEC type membrane in units of mechanical frequency where Ω = 15.2 kHz, *g*_0_ = 32.8 Ω and *J* = *π *Ω. In (**a**) the membrane’s oscillation amplitude is directly proportional to the number of photons in the cavity *N*. (**b**) shows that by evolving the system in the transparent membrane regime the number of photons in each of the cavities can be interchanged.

We plot the dynamics of the membrane under evolution of 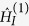 or 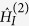 in Fig. 3 in the case of an initial product state of the membrane and optical fields to clearly demonstrate the driving and flipping of the membrane’s motion.

Now that a method of interchanging, or “flipping”, the state of the two cavity modes has been established the membrane’s displacement can be driven in the manner shown in Fig. 2. If the flipping process is repeated *N*_*F*_ times, whenever 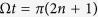 is satisfied, the membrane’s maximal displacement will increase linearly with *N*_*F*_. Ignoring dissipative effects, the unitary evolution can be expressed by,


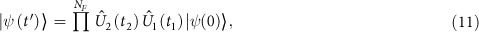


where 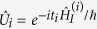 with 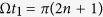 and 
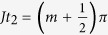
 for arbitrary integers *n*, *m*. Ideally we require *m* = *n* = 0 to reduce the evolution time and hence the effects of decoherence. Numerical simulations of this evolution will be performed in later sections where the effects of decoherence will be considered (see Section *Numerical Simulation*). This evolution will be the basis for both the creation and ‘growth’ of a cat state.

### Generation of a Cat State

While a possible mechanism for ‘growing’ the spatial extent of the mechanical cat state has been proposed, the cat state must first be created. One of the most common approaches to creating cat states is to first establish entanglement in the system. Once an entangled state is created there are many protocols which can be used to reduce the entangled state into a cat state. However, the majority of these protocols require that measurements are made on the system, projecting it into the cat state. This means that each of these protocols are probabilistic. In this section a similar approach to cat state creation will be made. We will first focus on establishing entanglement between the membrane’s position and the two cavity modes. This entanglement will be produced by preparing the system in specific initial states then evolving it in the manner discussed above. Several possible initial states will be considered with the intention of maximising the extent of the spatial displacement of the membrane’s position. The second half of this section will focus on disentangling the membrane from the two cavity modes to produce the desired cat state. Here, several possible disentanglement protocols which do not rely on measurement will be discussed with the use of the MITM model alone. Finally, an ensemble of atoms will be introduced into the MITM system to show that a completely deterministic and experimentally feasible disentanglement protocol is possible.

### Initial Conditions

As mentioned above, a common approach to cat state creation is to first establish entanglement. This means that a set of initial conditions must be determined which, when evolved under Eq. (11), leave the system in an entangled state that can be reduced into a cat state. In the proposed system this requires the generation of quantum entanglement between the membrane’s position and cavity modes. One possible approach to achieving this entanglement is to initialise the system in a state that corresponds to the membrane in its ground state while the cavity modes are in a NOON state^28^. In the MITM system the NOON state corresponds to a quantum superposition of the left cavity containing *N* photons while the right is empty and the right cavity containing *N* photons while the left is empty. The initial state can be expressed as,


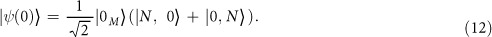


By evolving this state through the use of Eq. (11) the membrane’s position is simultaneously evolved under the two potentials depicted in Fig. 2. After such an evolution the state of the system is,


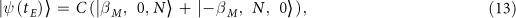


where *C* is a normalisation constant and *β*_*M*_ represents the coherent state amplitude of the membrane which depends on the number of ‘flips’ which have been applied as well as the number of photons in the NOON state, *N*. Typically NOON states are experimentally difficult to create for *N* > 2. From Eq. (7), it is clear that a large *N* is required to achieve large spatial displacements of the membrane. This means that, unless an ultra-small membrane is considered, an alternative set of experimentally feasible initial conditions is required.

There are two possible alternatives to initialising the cavities in a NOON state. These include (A) the coherent state analogue of the NOON state, referred to as an entangled coherent state (EC) and (B) the entangled squeezed-coherent state (ESC)^29^,^30^. For possibility (A): an EC can be produced in the proposed setup by initialising the left cavity in a coherent superposition state (CSS) and the right cavity in a coherent state then evolving the system in the highly transmissive regime, Eq. (8)^29^. Evolution under Eq. (8) for the correct duration effectively acts as a 50:50 beam splitter on the two input states. If the two cavities are prepared in the states 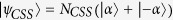 and 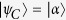 then evolved under the beam splitter dynamics the state,


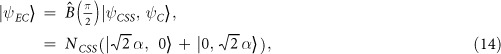


is generated, where *N*_*CSS*_ is a normalisation constant and 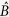 the beam splitter operator,


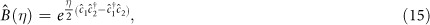


for arbitrary input modes 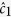 and 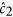. While this provides a coherent state analogue of the NOON state, the creation of EC states relies directly on the generation of 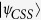. Such coherent superposition states have only been demonstrated experimentally with small coherent amplitudes, 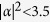, thus EC initial states with large coherent amplitudes may be beyond current experimental capabilities^12^,^31^.

For possibility (B): Entangled squeezed coherent states can also be generated using the proposed setup, Fig. 1. An ESC state can be created by preparing the left mode in a squeezed state and the right mode in a coherent state then evolving the system in the highly transmissive regime, Eq. (8). Both squeezed vacuum states and squeezed single photon states, corresponding to initialisation with a parametrically down converted photon, have been used to demonstrate the creation of such entangled states^32^,^33^. The resulting state can be expressed by,


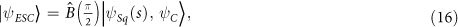


where the squeezed state is defined as 
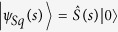
 with the squeeze operator,


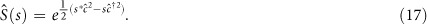


The appeal of ESC states is that they strongly resemble high *N* NOON states where there is essentially no limit on the size of *N*. The largest ESC states which have been created correspond to high NOON states with *N* ≤ 9 34. Thus, although they outperform EC states, ESC states are still far from the 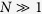 NOON-like states that are needed to displace large membranes. This means that realistically only very light membranes can be considered in our proposed cat state creation protocol.

### Disentanglement

As discussed in the previous section, to produce the desired cat state entanglement must first be established between the membrane’s position and the cavity modes. This entanglement can be achieved by initialising the system in the state Eq. (12) and performing the evolution described by Eq. (11). However, this evolution leaves the system in the state, Eq. (13), where the membrane’s reduced position is in a completely mixed state. To convert Eq. (13) into a pure cat state of the membrane’s position the membrane must be disentangled from the two cavity modes.

Disentangling is typically is a very difficult task. It is sometimes possible to find a unitary operation that will deterministically disentangle systems but such operations require the application of conditional quantum gates and these are typically extremely hard to implement physically. Rather than attempt unitary disentanglement many protocols implement probabilistic measurement based methods for disentanglement. In what follows we initially explore a naive approach to disentangle the photonic and motional systems by letting the photons escape from the optical cavities. This method fails for the reasons we outline below. We then discuss a unitary operation which achieves deterministic disentanglement by allowing the membrane’s reflectivity to change depending on the membrane’s displacement. Although formally this approach works there are currently no experiments capable of controlling transparency over pico-nano meter scales. Guided by this we note that methods to unitarily disentangle discrete (e.g. spin), systems are physically more tractable and we suggest introducing a cold atomic gas comprised of three level atomic systems and describe a protocol which converts the membrane-optical mode entanglement into membrane-atomic excitation entanglement. Once in the latter form we are then able to more easily perform the necessary conditional operations on the atomic-membrane system to unitarily disentangle them. The resulting protocol is complex but shows that deterministic unitary disentanglement is possible.

As mentioned above, a naive approach to disentangle the state Eq. (13) involves simply allowing the N cavity photons to decay from both cavities, potentially evolving the system into the disentangled state 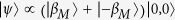. This approach, however, is flawed as, in fact, the final state of the membrane is a mixed state due to the non-unitary evolution associated with the photon loss.

A second approach is a completely unitary, deterministic, method to disentangle the membrane’s position from the cavity photons. To achieve this we must posit the ability to conditionally flip the state of the cavity modes, depending on the membrane’s position. That is, performing the transformation,


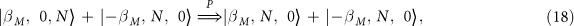


which can be achieved by the unitary operation,


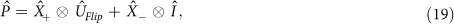


where 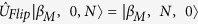, and 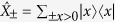 are position projection operators onto the positive/negative membrane position axes. While this approach has the potential to deterministically disentangle the state, it essentially requires that the transmission rate of the membrane is also position dependent, *J*(*t*, *x*), which is extremely experimentally challenging for pico to nano-metre displacements of the membrane. If such control over the membrane’s transmission rate were possible our disentanglement discussion would end here.

**Figure 4:**
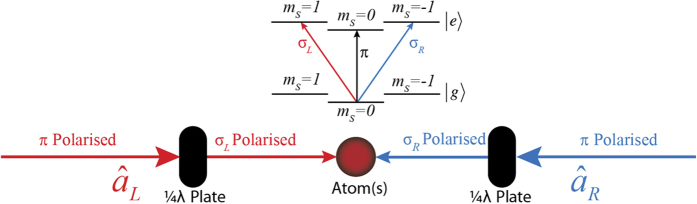
Depiction of the transfer of entanglement from the cavity modes to an ensemble of atoms. The left and right cavity modes are left/right hand circularly polarised then directed into a cloud of atoms. Two separate transitions are excited depending on the polarisation, shown above.

As the two disentanglement methods discussed above either fail or are currently infeasible an alternative disentanglement protocol for Eq. (13) must now be established. As mentioned above, we proceed by transferring the excitations held in the photonic subsystem to a new discrete atomic ensemble subsystem containing *M* > *N* three-level atomic atoms . We achieve this transfer via tagging the photons in the two optical cavities in the MITM system via separate polarizations and using this polarization degree of freedom to excite polarization dependent transitions in the atomic ensemble. Once encoded into the atomic ensemble we describe a relatively simple method to perform the disentanglement we seek by manipulating the internal states of the atomic ensemble. The entanglement transfer can be achieved if the light in the left/right cavities is left/right hand (*σ*_*L*_/*σ*_*R*_) circularly polarised. By quickly introducing an ensemble of *M* 3-level atoms into the system the photons in the left/right cavity modes are separately absorbed into the *m*_*s*_ = ±1 excited states of the atoms. This process simultaneously removes the photons from both cavities while encoding the state of each cavity into the internal excited states of the ensemble of atoms, see Fig. 4. To explore the mapping of the entanglement to the atomic ensemble the case of a single 3-level atom interacting with the *σ*_*L*_/*σ*_*R*_ polarised photons can be considered. In this case the interaction is described by the Hamiltonian,


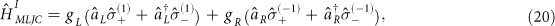


where the detuning has been set to zero and 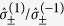 are the atomic raising and lowering operators associated to the degenerate 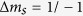 transitions with coupling strengths *g*_*L*_/*g*_*R*_. If the interaction strengths are identical this Hamiltonian can be easily extended to account for *M* atoms. By incorporating *M* atoms into the entangled state, Eq. (13), and evolving it under the *M*-atom extension of Eq. (20) for a specific time *t*_3_, ensuring all photons are absorbed, the entanglement is essentially transferred from the two cavity modes to the *M* atoms,


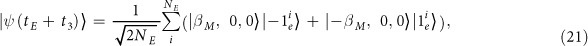


where 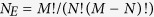, 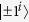 denotes the *i*^th^ permutation of the *N m*_*s*_ = ±1 magnetic states over the *M* atoms and the subscripts *e*,*g* the optical state, see Fig. 4.

This evolution requires 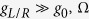 to avoid the effects of the changing photon pressure force on the membrane. If this condition is not met, the amplitude of the membrane’s oscillation will decrease due to the decreasing number of photons in the cavity. It is also important to note here that if the cavity is initialised in an EC state, to maintain determinism, this transfer of entanglement requires that 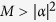. The membrane-atom entangled state, Eq. (21), can now be disentangled by applying a *π* pulse of linearly polarised light to the atoms. As linearly polarised light is comprised of both left and right hand circularly polarised light, application of a *π* pulse will simultaneously send the *N m*_*s*_ = ±1 state atoms to the ground state. The remaining *M* − *N* atoms will be excited into the *m*_*s*_ = 0 optically excited state. After application of this pulse Eq. (21) reduces to,


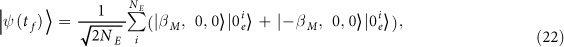


which is a pure cat state of the membrane’s position. In this case 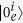 denotes the *i*^th^ permutation of the *M* − *N m*_*s*_ = 0 optically excited magnetic states over the *M* atoms.

### Schematic of Experimental Realisation

In the previous sections it was shown that, in theory, a cat state of the membrane’s position can be deterministically created by controlling the membrane’s transmission rate and selectively introducing *M* atoms into the system. This section will focus on illustrating the cat state creation protocol with a schematic of an optically based experiment capable of both achieving the same effective optical dynamics as modulating the transmission rate of the membrane and the introduction of an ensemble of atoms.

The first task in our cat growth protocol we address is to alter the membrane’s transparency with time. When the membrane is transparent the optical fields in the two MITM cavities interchange (flip) and this flipping is an essential component in our protocol. Controling the membrane transparency in time is experimentally challenging and so we propose an alternative method to interchange the optical fields between the two Fabry-Pérot (FP) optical cavities in the MITM setup. We will show that one can effectively rapidly switch between two setups: (A) the membrane located in a Fabry-Pérot cavity (traditional MITM), and (B) the membrane located in a ring cavity that envelops the Fabry-Pérot arrangement in (A) . By switching the setup into (B) for a short duration and then back to (A) we can effectively provide an optical route to interchange the optical fields in setup (A) without having to control the membrane’s transparency.

The second task, that of quickly inserting an atomic ensemble and allowing interaction between the ensemble and photons from both FP cavities in setup (A), is again achieved through switching the setup into a ring cavity which now contains the atomic ensemble.

**Figure 5:**
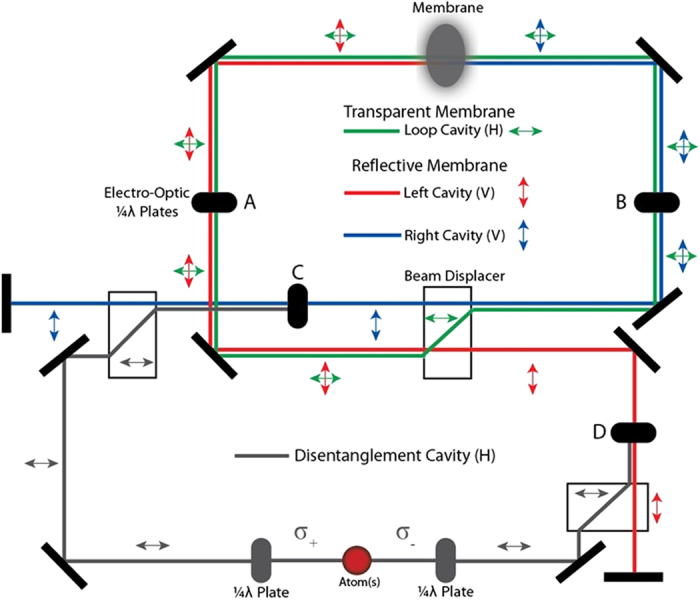
Suggested schematic experimental configuration for effective flipping of the MITM optical cavity fields and the disentanglement operation. The three cavities necessary for both the preparation and disentanglement of the cat state are colour coded. The original MITM setup where the membrane separates two Fabry-Pérot cavities is colour coded via blue and red optical paths with vertically polarized photons (shown by vertical double arrows). The Electro Optic Quater Wave Plates [EOP] (A, B) are used to rapidly switch the polarisation of the light fields to horizontal polarization. Due to the presence of polarization dependent beam displacers, horizontally polarized light propagates in a ring resonator (green optical path), and this evolution is left on for the brief duration required to flip the optical fields on either side of the membrane. Then the EOPs (A, B) are switched back to return to the original Fabry-Pérot MITM setup. When finally one wishes to disentangle the membrane and optical fields EOPs (C, D) are used to route the photons into another ring resonator that also includes an atomic ensemble.

Both of the above tasks are implemented via polarization control of the optical paths taken by the photons which we now describe in more detail. The schematic experiment achieves the first task, interchanging the optical consists of a multi-cavity system where each cavity is distinguished by the polarisation of the light in the system, shown in Fig. 5. Each of the cavities are separated through the use of appropriately positioned beam displacers and alternated between by the activation of Electro-optic quarter wave plates (EOP). For vertically polarised light, the cavity resembles that of a Fabry-Pérot cavity where the membrane is positioned in the center. The Fabry-Pérot cavity is shown in Fig. 5 by red and blue lines, each corresponding to the left/right cavities. This means that if the light in the system is vertically polarised and the natural transmission rate of the membrane satisfies 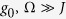 the system can be approximately described by Eq. (6). If the light is horizontally polarised the system corresponds to a Ring cavity containing the membrane, shown as green in Fig. 5. In this case the effective transmission rate of the membrane drastically increases as the light can freely travel between the left/right cavities and hence the system can be approximately described by the highly transmissive Hamiltonian, Eq. (8). The evolution described by Eq. (11), which is required to drive the displacement of the membrane, could be realised by alternating between these two cavities through the activation of the EOPs *A* and *B* in Fig. 5. Introduction of many atoms into the system could be achieved by introducing a third cavity using a similar technique as above. At the end of the evolution, Eq. (11), the system remains in the highly reflective regime. If at this time the EOPs *C* and *D* in Fig. 5 are activated the system will again resemble a Ring cavity, but one which contains *M* atoms as well as the membrane. Provided that the atoms are each prepared in the optical ground state, Δ*m*_*s*_ = ±1 transitions will be excited as the light of each mode is left/right hand polarised by two separate quarter wave plates, entangling the membrane with the atomic states.

To create a cat state with the suggested experiment a specific operational protocol must be followed. Once the system has been initialised in the state Eq. (12) with *M* ground state atoms the experiment starts with an evolution in the highly reflective regime for time *t*_1_. This evolution corresponds to driving the membrane to the maximal possible displacement in the initial potential well. We predict the time at which this maximal displacement will occur. Then, at this predicted time, we switch the system into the effective transparent membrane setup to begin the dynamics that will interchange the optical fields in the cavities. At no instant are we allowed to perform any measurement of the membrane’s position or cavity field as such a measurement will cause a collapse of the cat state superposition we wish to achieve. To switch the system into the transparent membrane regime the EOPs A and B in Fig. 5 are activated.

Evolution in this regime for time *t*_2_ will flip the state of the cavity modes, which corresponds to displacing the harmonic potential. As it was shown that after time *t*_2_ the occupation of the cavity modes will be completely interchanged, see Eq. (9). Deactivating these EOPs will then switch the system back into the high opacity regime but under a displaced harmonic potential, increasing the potential energy of the membrane. By evolving again in this regime for time *t*_1_, the membrane’s maximal displacement will increase in accordance with Eq. (7). This process can be repeated until the desired spatial displacement is acquired. Once the desired displacement is achieved the system will reside in the high opacity regime in the entangled state, Eq. (13). In order to create a cat state, the membrane must now be disentangled from the cavity modes. By activating EOPs C and D in Fig. 5, *M* atoms can be introduced into the system. Evolving this combined system for time *t*_3_ will transfer the entanglement between the membrane and the cavity modes to the atoms, as shown in Eq. (21). Finally, by application of a linearly polarised *π* pulse to the atomic ensemble, a cat state of the membrane’s position can be produced.

**Figure 6:**
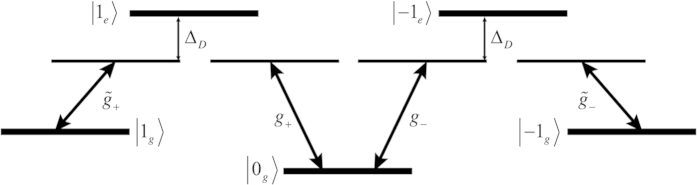
Depiction of the Raman transition which can be applied to the 3-level atomic system to excited the magnetic levels of the optical ground state, 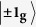, rather than those of the optically excited state, 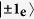
. The interaction strength for each transition is denoted by the respective *g*.

As optical transitions are considered in the schematic experiment, application of a *π* pulse to the ensemble of 3-level atoms to disentangle the system is quite difficult experimentally. This difficulty stems from the short lifetimes of the optically excited states, in turn, making the disentanglement process experimentally challenging. However, the optically excited states of the 3-level atoms can be bypassed through the use of a Raman transition. To do so, we must consider atoms with triplet optical ground and excited states. Such atoms can be directly excited into the *m*_*s*_ = ±1 levels of the optical ground state by detuning the left/right optical cavity modes from the atomic transition and introducing a similarly detuned coherent field, depicted in Fig. 6. This is beneficial as these levels of the optical ground state are significantly longer lived, allowing for a more practical implementation of the *π* pulse to the atomic ensemble. Bypassing the optically excited state requires that both the left/right optical cavity modes and the classical field are off resonance with the atomic transition such that the detuning, Δ_*D*_, satisfies 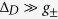, where *g*_±_ denotes the coupling strength to the *m*_*s*_ = ±1 transitions, shown in Fig. 6. An effective Hamiltonian for this process can be derived by time-averaging the dynamics of the system. For an interaction picture Hamiltonian of the form,


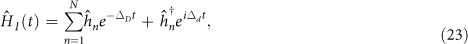


the effective Hamiltonian can be expressed as^35^,


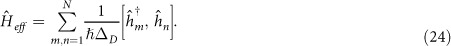


In the case of a single 3-level atom and a single, left hand circularly polarised optical mode, shown in the left half of Fig. 6, the interaction picture Hamiltonian of the system before time-averaging is,


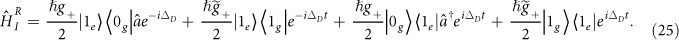


An identical Hamiltonian can also be written to describe a right hand circularly polarised optical mode, corresponding to the 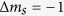 transition, depicted in the right half of Fig. 6. Through the use of Eq. (24) the effective Hamiltonian for a single atom system coupling to both left and right hand circularly polarised optical modes is 
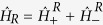
 where,


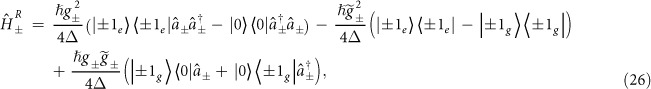


with 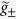 denoting the coupling rate associated with the transition between the virtual *m*_*s*_ = ±1 levels of the optical excited state and *m*_*s*_ = ±1 levels of the optical ground state, shown in Fig. 6. This Hamiltonian can be easily extended to account for *M* atoms. The discussed Raman transition optically encodes the cavity information into the optical ground triplet states of the atoms. The atomic ensemble can then be disentangled from the membrane’s position by application of a microwave *π* pulse.

### Numerical Simulations

Both the theory and a possible experimental realisation of the cat state creation protocol have now been described. Simulations of the proposed protocol can now be performed to determine the quality and size of the resulting cat states. In this section numerical simulations of the membrane’s dynamics with the consideration of dissipative effects will first be performed to show that large displacements of the membrane can be achieved. Throughout the simulations a BEC consisting of ~10^5^ Rubidium-87 atoms will be used due to its small mass and its compatibility with the MITM model^18^. The two cavity modes will then be initialised in a NOON state, Eq. (12), to show that, after evolution and application of the disentanglement protocol, a cat state can be created.

**Figure 7:**
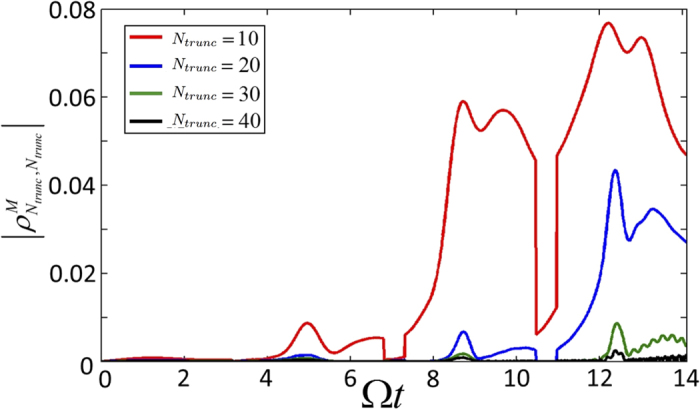
Graph to monitor accuracy of numerics due to finite truncation of the membrane’s Hilbert space dimension. In the numerics we restrict the available Fock states that describe the quantum state of the membrane’s position to have a Fock number *n* ≤ *N*_*trunc*_. We monitor the extreme most value of the membrane’s reduced density matrix, 
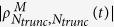
, during the time evolution of the protocol. We must choose *N*_*trunc*_ large enough to avoid significant population in this element and find that we must have *N*_*tunc*_ ~ 40.

**Figure 8:**
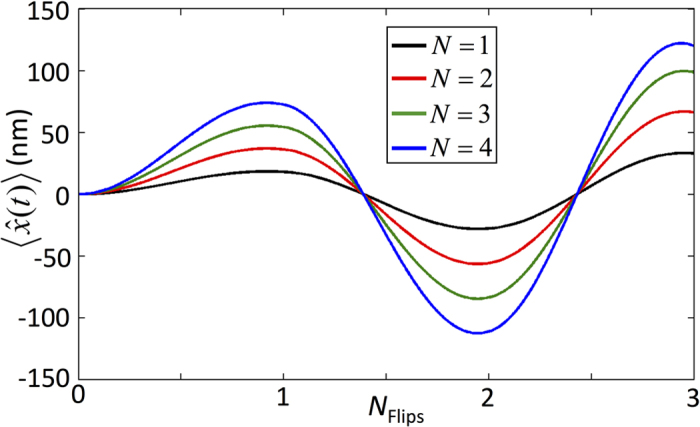
The dynamics of a BEC’s position when the system is initialised in the state 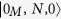
. Several values of the photon number, *N*, were used and dissipative effects were considered. The simulations were performed in units of Ω with *g*_0_ = 32.8Ω, *κ*_*c*_ = 0.17Ω and 
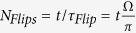
.

The dynamics of the system can be simulated by solving the full master equation,


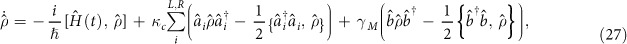


where the time dependence in the Hamiltonian describes the alternation between the two different opacity regimes, Eq. (6) and Eq. (8). Here the parameters *κ*_*c*_ and *γ*_*M*_ denote the cavity and mechanical damping rates respectively. Before continuing, it will be assumed that the mechanical damping rate, *γ*_*M*_, is negligible with respect to the optical damping rate during the time scales that will be considered, 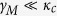
19. The above master equation was solved using MATLAB’s Quantum Optics Toolbox (QOT). The alternation between the reflective/transparent regimes can be simulated by performing successive simulations under each regime. We explored the accuracy of our numerics using various indicators. Both Tr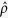 and the traces of the reduced systems were unity to within the absolute and relative error (10^−8^), of the integrator in QOT. A more important source of numerical error arises from truncating the Hilbert spaces of the cavities and membranes. Analytically each of these are infinite dimensional but in the numerical simulation we truncate the individual cavity Hilbert spaces to *n* ≤ 4, while the membrane’s Hilbert space is truncated to *n* ≤ *N*_*trunc*_. The cavity Hilbert space truncation is found to be adequate while in Fig. 7 we plot the time dependence of the largest Fock element of the reduced density matrix of the membrane: 
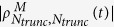
. We must choose a Fock state truncation *N*_*trunc*_ large enough to avoid significant population in this element during our simulation and we set *N*_*trunc*_ = 40. By solving Eq. (27) with the initial condition 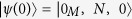 the driving of the membrane’s displacement can be demonstrated, see Fig. 8. To examine the dependence of the photon number difference, Δ, on the maximal displacement, simulations were performed using several experimentally achievable values of *N*. A linear increase in the maximal displacement with *N*, predicted by Eq. (7), can be clearly observed in Fig. 8 where the mechanical frequency was set to 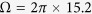 kHz, the mechanical coupling rate to 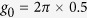 MHz, the cavity damping rate to 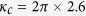 kHz^19^, with a BEC of mass *m* = 17.3 ag^18^. The results also show that nanometre displacements of the BEC’s center of mass position from the origin can be attained with only three flips of the two cavity states. While the results show that under these conditions initialising the cavity modes in small number states is somewhat effective for displacing the BEC, many more flips are required to achieve large spatial displacements of more massive membranes. Under these conditions performing more than five flips is not possible if the cavities are initialised in small number states as, in this case, the cavity damping rate is on the order of the mechanical frequency (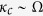). This means that by the time the BEC reaches its maximal displacement a significant portion of the photons are lost from the cavity. The loss of photons from the cavity also produces a short time delay between the point in which the membrane achieves its maximal displacement and the application of the flip. This is most easily explained in the displaced harmonic oscillator picture, Fig. 2. As the system evolves photons are lost from the cavities causing the center of the two potential wells to shift towards the origin. This results in the achievement of maximal displacements at times slightly shorter than those predicted in Eq. (7). The times predicted by Eq. (7) were used in these simulations to demonstrate this effect as realistically these short time delays must be accounted for.

**Figure 9:**
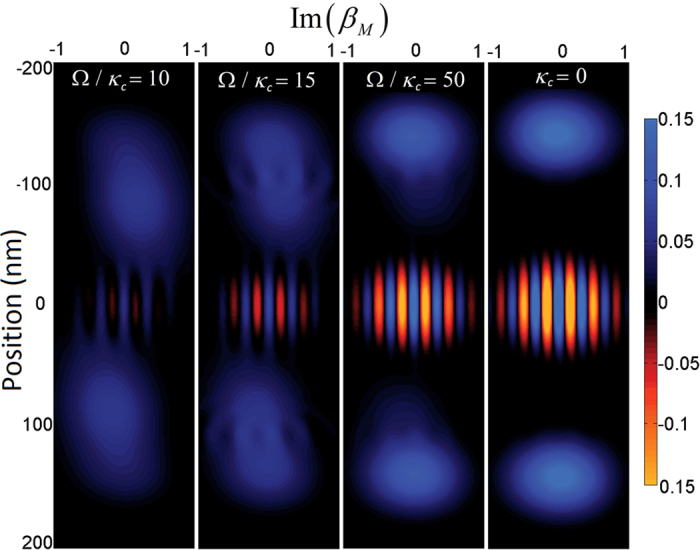
Density plots of the Wigner function for different cavity damping rates, *κ*_*c*_. These simulations were performed using the parameters for a BEC shown above with *N* = 2 photons in the system and *N*_*Flips*_ = 3. The negativity of the Wigner function shows that the final state is still a non-classical state^36^.

In order to create a cat state of the membrane’s position the cavity must be initialised in a NOON state, Eq. (12). In what follows the cavity will be initialised in a NOON state with *N* = 2. Of course, evolving this initial state results in 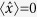, as the membrane is simultaneously displaced in both the + *x* and −*x* directions. Two approaches will be made to observe the cat state produced. The first involves the calculation of the Wigner function to observe the ‘quantumness’ (negative regions of the Wigner function) of the final state while the second involves calculating the fidelity between the cat state produced and a corresponding ideal cat state. Beforehand, however, a simplification can be made to the disentanglement process to increase the efficiency of the simulations. As the time scales of the disentanglement procedure are required to be significantly shorter than that of the standard evolution which drives the membrane’s displacement, 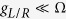, losses during this procedure will be neglected. This also means that the disentanglement protocol can effectively be performed by application of the disentanglement unitary described in Eq. (19). Simulations of the disentanglement protocol are essentially those of the Tavis-Cummings model with *M* identical atoms and two modes of light. A cat state of the membrane’s position can then be produced by application of the disentanglement unitary after the entire evolution has been made,


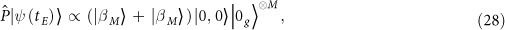


where 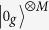 describes the state in which all *M* atoms are in the optical ground state. The resulting Wigner function of the membrane’s state after application of 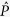 is presented in Fig. 9 for several cavity damping rates. These results show that the final state strongly resembles that of a typical cat state with decoherent effects similar to those observed by Haroche^12^. They also show that, if the ratio between the trap frequency of the BEC and the cavity loss rate can be increased by a factor of ~10, spatial separations of up to 300 nm can be achieved between the two center of mass positions of the 10^5^ atom BEC. This is more than double the displacement achieved in the entangled state analogue where only a single ion was used^11^. To determine the degree of resemblance between the final state, 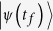, and a typical cat state 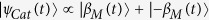, the fidelity can be calculated,


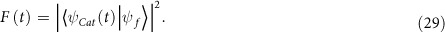


**Figure 10:**
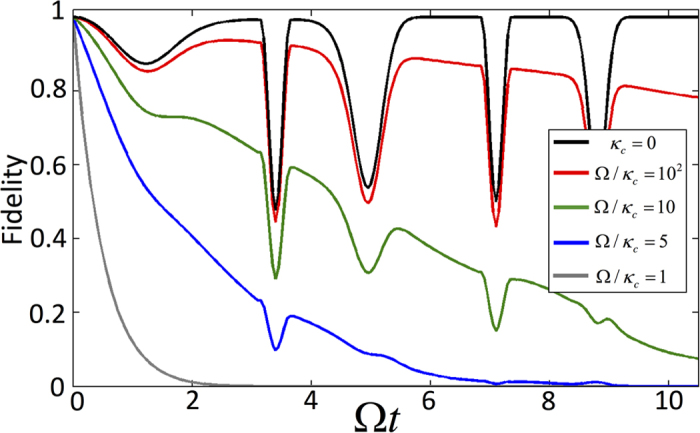
The fidelity between the evolved state at each time step with a corresponding ideal cat state using several cavity dampening rates, *κ*_*c*_, and *N* = 2. The sharp peaks correspond to evolution in the transparent membrane regime whereas the thicker peaks correspond to an artefact of disentanglement unitary, as the projection is only conditional on position and not momentum. The fidelity is unity initially as 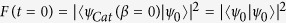.

The coherent amplitude, *β*_*M*_(*t*), corresponds to the membrane’s state when evolving 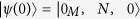 under identical conditions. The results show (Fig. 10) that with 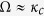 the cat state is destroyed before the first flip is performed. Using literature values, 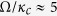, the state partially survives the first cavity state flip^19^. For ideal results, *F* > 80%, the ratio between the mechanical trap frequency of the BEC and the cavity loss rate must be increased by a factor of 20 where the cat state survives all three cavity state flips.

## Discussion

In this work a quantum system designed for the deterministic creation of macroscopic quantum states is proposed. The system consists of a BEC type membrane which is placed inside a Fabry-Pérot type optical cavity. It is shown that by controlling the opacity of the BEC its displacement from the origin can be driven at a rate proportional to the number of photons in the system. This result was then used to produce and essentially grow the spatial extent of a cat state of the BEC’s position. This required the initialisation of the two cavities in a NOON like optical state, which was shown generates entanglement between the membrane’s position and the cavity modes after the system is evolved. To reduce this entangled state to a cat state a deterministic disentanglement procedure was proposed which involved the transfer of the entanglement to an ensemble of atoms. The cat state creation protocol was then illustrated using a schematic of an experimental platform which was capable of both effectively flipping the cavity optical states and applying the deterministic disentanglement procedure.

Finally, several simulations were performed showing that large spatial displacements of the BEC could be achieved using only a small number of photons in the system. Simulations of cat state creation were also performed which showed that relatively high fidelity cat states could be produced if either slightly larger mechanical frequencies or slight smaller cavity loss rates than those achieved in previous experiments could be reached. Overall the proposed cat state creation protocol provided an experimentally feasible method of deterministically creating cat states significantly larger than the majority of previous proposals. Creation of such states are essential to further the understanding of quantum decoherence and have many potential applications in a wide range of quantum technologies.

## Additional Information

**How to cite this article**: Lombardo, D. and Twamley, J. Deterministic Creation of Macroscopic Cat States. *Sci. Rep.* **5**, 13884; doi: 10.1038/srep13884 (2015).
